# Cell-specific regulation of proliferation by Ano1/TMEM16A in breast cancer with different ER, PR, and HER2 status

**DOI:** 10.18632/oncotarget.18662

**Published:** 2017-06-27

**Authors:** Huizhe Wu, Hui Wang, Shu Guan, Jing Zhang, Qiuchen Chen, Xiaodong Wang, Ke Ma, Pengfei Zhao, Haishan Zhao, Weifan Yao, Feng Jin, Qinghuan Xiao, Minjie Wei

**Affiliations:** ^1^ Department of Pharmacology, Liaoning Key Laboratory of Molecular Targeted Anti-Tumor Drug Development and Evaluation, China Medical University, Shenyang, 110122, P.R. China; ^2^ Department of Ion Channel Pharmacology, School of Pharmacy, China Medical University, Shenyang, 110122, P.R. China; ^3^ Department of Breast Surgery, The First Hospital of China Medical University, Shenyang, 110001, P.R. China

**Keywords:** Ano1, TMEM16A, Ki67, breast cancer, proliferation

## Abstract

The calcium-activated chloride channel Ano1 (TMEM16A) is overexpressed in many tumors. However, conflicting data exist regarding the role of Ano1 in cell proliferation. Here, we performed immunohistochemistry to investigate the expression of Ano1 and Ki67 in 403 patients with breast cancer, and analyzed the association between the expression of Ano1 and Ki67 in breast cancer subtypes categorized according to estrogen receptor (ER), progesterone receptor (PR), and human epidermal growth factor receptor 2 (HER2). Ano1 expression was negatively correlated with Ki67 expression. Ano1 overexpression more frequently occurred in ER-positive or HER2-negative patients with the low expression of Ki67. Ano1 overexpression was associated with longer overall survival (OS) in breast cancer with the low expression of Ki67, especially in ER-positive, PR-positive, and HER2-negative breast cancer. Multivariate Cox regression analysis showed that Ano1 overexpression was a prognostic factor for longer overall survival in ER-positive, PR-positive, or HER2-negative patients with the low expression of Ki67. Furthermore, Ano1 promoted cell proliferation in ER-positive, PR-positive, and HER2-negative MCF7 cells, but inhibited cell proliferation in ER-negative, PR-negative, and HER2-negative MDA-MB-435S cells. Our findings suggest that Ano1 may differentially regulate cell proliferation in a subtype of breast cancer defined by ER, PR, and HER2. Combined expression of Ano1 and Ki67 may be used for predicting clinical outcomes of breast cancer patients with different subtypes of ER, PR, and HER2.

## INTRODUCTION

Anoctamin 1 (Ano1, TMEM16A) is a novel calcium-activated chloride channel (CaCC) identified by three independent laboratories in 2008 [1-3]. Ano1 is involved in a variety of physiological functions including epithelial secretion, smooth muscle contraction, neuronal and cardiac excitation, and pain [4-7]. Ano1 has been implicated in many diseases such as cancer, hypertension, and cystic fibrosis [4, 8, 9]. Recently, Ano1 modulators with a relative high selectivity have been developed [10-12], and are believed to be potentially used for the treatment of these diseases [4].

Before the discovery of Ano1 as a CaCC, Ano1 has been found to be overexpressed in many cancers including gastrointestinal stromal tumor, esophageal squamous cell cancer, and head and neck squamous cell carcinoma (HNSCC) [13-16]. Ano1overexpression is also found in many other tumors including breast cancer [17-19], prostate cancer [20], pancreatic ductal adenocarcinoma [21], gastric cancer [22], and chondroblastoma [23]. Amplification of the corresponding chromosomal region in 11q13 contributes to Ano1 overexpression in many cancers including breast cancer [18, 24-26]. However, the 11q13 amplification only occurs in approximately 15% of breast cancer patients [27]. It is unlikely that the 11q13 amplification is the only contributor to Ano1 overexpression in breast cancer. Other regulatory mechanisms that are responsible for Ano1 overexpression are expected to exist. It has been reported that the inhibition of histone deacetylase (HDAC) downregulated Ano1 expression in breast cancer cells, suggesting that epigenetic regulation of Ano1 by HDAC may contribute to Ano1 overexpression in breast cancer [28]. Ano1 has been found to be downregulated via promotor methylation in metastatic nodal tissues from HNSCC patients [29]. Our previous study have shown that in ER-negative breast cancer, Ano1 expression was higher in PR-positive tumors than in PR-negative tumors, suggesting that Ano1 expression may be regulated by the ER/PR signaling pathway [19].

Although Ano1 has been found to regulate cell proliferation, the role of Ano1 in cell proliferation has not been completely understood [4]. Ano1 overexpression promotes cell proliferation in tumors such as HNSCC, breast cancer, glioma, and prostate cancer [18, 20, 24, 30, 31], as well as interstitial cells of Cajal [32] and renal cyst-forming epithelial cells [33]. However, cell proliferation is not reduced in pancreatic ductal adenocarcinoma cells and gastric cancer cells treated with Ano1 shRNAs or inhibitors [21, 22]. In addition, overexpression of various Ano1 isoforms identified in breast cancer cells does not affect cell proliferation in HEK-293 cells [17]. The inhibitory effect of Ano1 on cell proliferation has been found in vascular smooth muscle cells. Ano1 overexpression inhibits angiotensin II-induced proliferation in vascular smooth muscle cells via Kruppel-like factor 5, which prevents activation of the Ano1 promoter by myocardin [34, 35]. It appears that Ano1 plays different roles in cell proliferation in different cells via cell type-dependent mechanisms.

Breast cancer is a heterogeneous disease that is clinically categorized into different subtypes based on estrogen receptor (ER), progesterone receptor (PR), and human epidermal growth factor receptor 2 (HER2) [36]. In this study, we investigated the expression of Ano1and Ki67, a well-known marker for cell proliferation, in subgroups of breast cancer patients, categorized according to the ER, PR, and HER2 status. The purpose of this study was to explore the association of Ano1expression with Ki67 expression in different subtypes of breast cancer, to identify the prognostic role of Ano1 in breast cancer with different subtypes, and to explore the possible cell-specific mechanisms of Ano1 overexpression in cell proliferation.

## RESULTS

### Clinicopathological characteristics

Table 1 summarizes clinicopathological charac-teristics of 407 breast cancer patients. The median age of these patients was 51 years (range, 20-82 years). Most patients (83.8%) did not have a family history of breast cancer. The majority of these patients had a tumor with >2 cm in size, histological Grade 2, and Stage I or II. Of the 407 patients, 278 (68.3%), 275 (67.6%), and 96 (23.6%) patients were ER-negative, PR-negative and HER2-positive, respectively. Triple-negative breast cancer (TNBC) patients were only recorded in 63 (15.5%) of 407 patients. Of the 407 patients, 358 patients received anthracycline-based chemotherapy alone or in combination with paclitaxel, and 316 patients received tamoxifen treatment.

**Table 1:** Clinicopathological baseline characteristics

Parameters	Cases	
	n	%
**Total n**	407	100
**Median age [range], years**	51[20-82]	
**Age (years)**		
<51	219	53.8
≥51	188	46.2
**Menopausal status**		
Premenopausal	213	52.3
Postmenopausal	194	47.7
**First-degree family history of breast cancer**		
No	341	83.8
Yes	66	16.2
**Tumor size (cm)**		
≤ 2.0	138	33.9
> 2.0	269	66.1
**Histological grade**		
Grade 1	46	8.0
Grade 2	325	56.3
Grade 3	36	6.2
**Clinical stages**		
I or II	290	71.3
IIIA∼IIIC	117	28.7
**Lymph node metastasis**		
Node-negative	199	48.9
Node-positive	208	51.1
**ER status**		
Negative	129	31.7
Positive	278	68.3
**PR status**		
Negative	132	32.4
Positive	275	67.6
**HER2 status**		
Negative	311	76.4
Positive	96	23.6
**Triple negative status**		
Non-triple negative	344	84.5
Triple negative	63	15.5
**Tamoxifen treatment**		
No	91	22.4
Yes	316	77.6
**Postoperative therapeutic regimens**		
Anthracycline alone or combined with paclitaxel	358	88.0
Other chemotherapies or treatments	49	12.0

**Abbreviations:** ER, Estrogen receptor; PR, Progesterone receptor; HER2, Human epidermal growth factor receptor.

Other chemotherapies included paclitaxel alone (*n*=21), NP regimen (navelbine plus cisplatin, *n*=15), TP regimen (Docetaxel plus cisplatin, *n*=11).

The patients were followed up for 5-71 months. Relapses occurred in 37 (9.1%) of 407 cases and breast cancer-associated deaths in 49 (12.0%) of 407 cases. The 5-years survival rate was 86.9%. The mean OS and DFS were 66 months and 63 months, respectively.

### The expression of Ano1 and Ki67 in breast cancer

**Figure 1:**
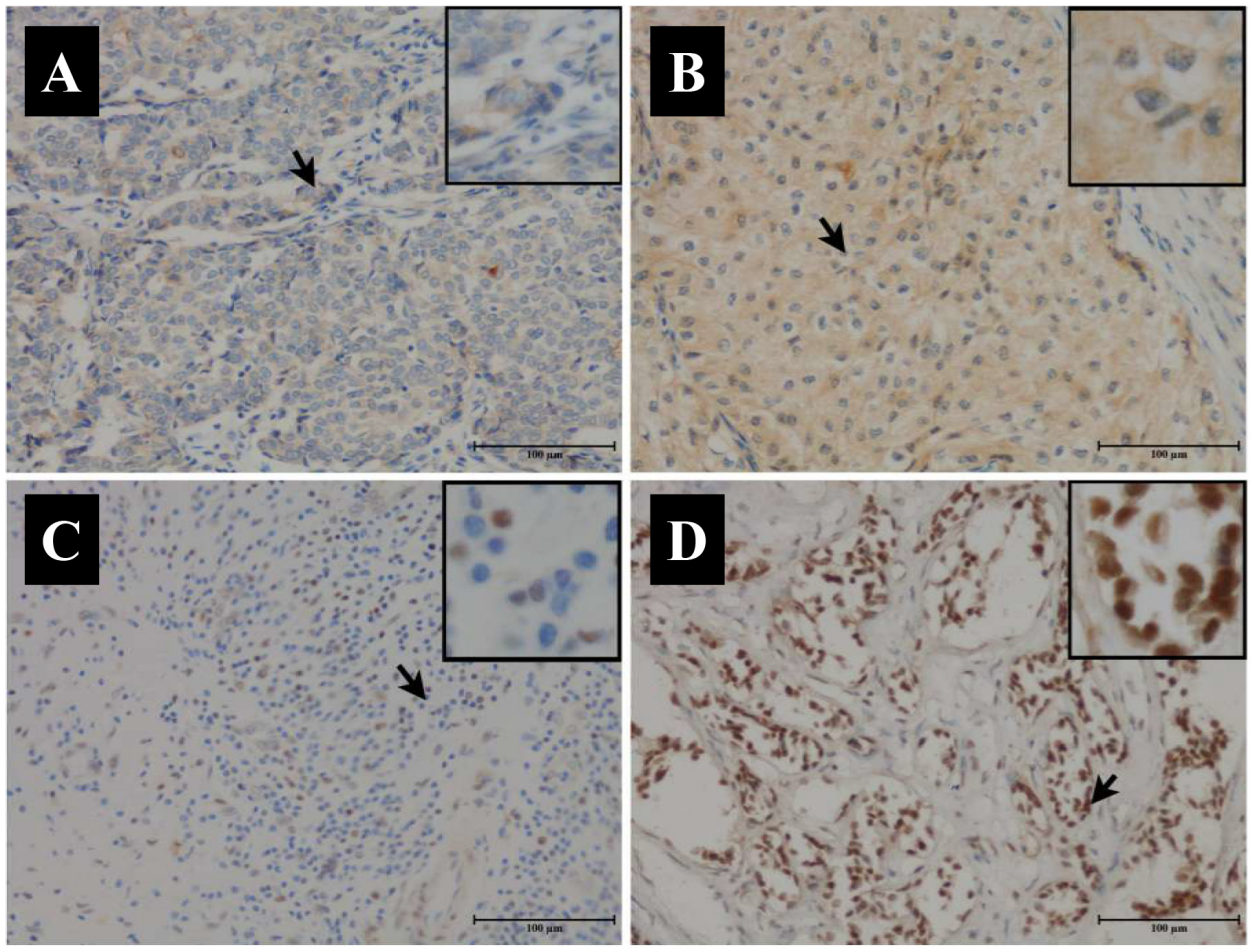
Representative immunohistochemical micrographs for the low **(A, C)** and high **(B, D)** expression of Ano1 (A, B) and Ki67 (C, D) in breast cancer samples. Arrows indicate the magnified regions in the insert. Magnification: ×100. Scale Bars: 100μm.

**Figure 2:**
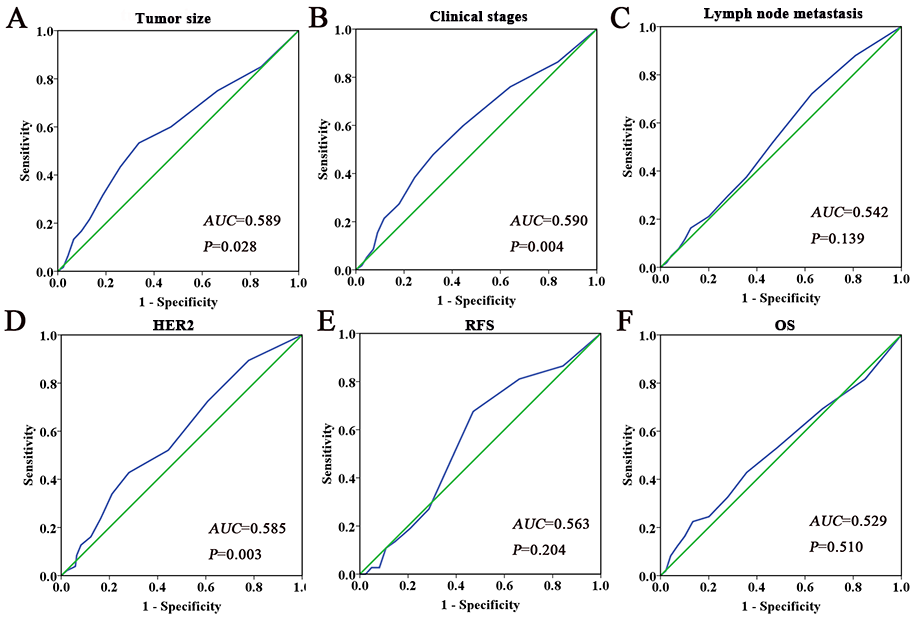
ROC curves were used to determine the cutoff score for the expression of Ano1 in breast cancer patients The sensitivity and specificity for histological grade **(A)**, clinical stage **(B)**, lymph node metastasis **(C)**, PR status **(D)**, TNBC status **(E)**, Ki67 status **(F)**, tamoxifen treatment **(G)**, and OS **(H)** were plotted for the expression of Ano1. The areas under curves (AUC) and *P* values were indicated.

**Figure 3:**
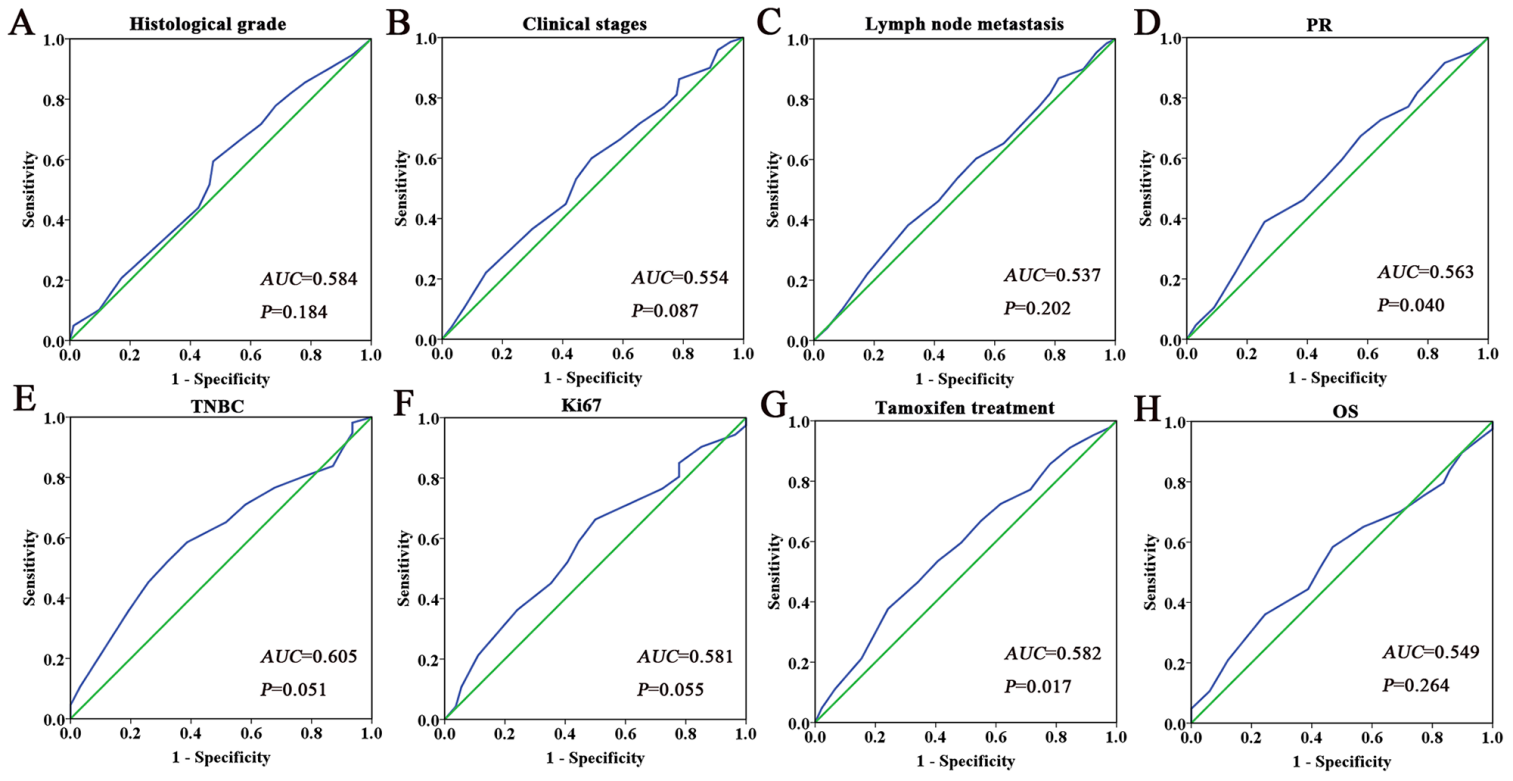
ROC curves were used to determine the cutoff score for the expression of Ki67 in breast cancer patients The sensitivity and specificity for tumor size **(A)**, clinical stage **(B)**, lymph node metastasis **(C)**, HER2 status **(D)**, RFS **(E)**, and OS **(F)** were plotted for the expression of Ano1. The areas under curves (AUC) and *P* values were indicated.

We performed immunohistochemistry to investigate the expression of Ano1 and Ki67 in 407 breast cancer samples (Figure 1). The optimal cutoff score for the expression of Ano1 and Ki67 was determined by ROC curves, based on the sensitivity and specificity for each clinicopathological feature of the 407 patients (Figures 2 and 3). ROC curves showed that the expression level of Ano1 was discriminated by PR (*P*=0.040), TNBC (*P*=0.051), and tamoxifen treatment (*P*=0.017) (Figure 2), and the expression level of Ki67 was discriminated by tumor size (*P*=0.028), clinical stages (*P*=0.004), and HER2 status (*P*=0.003) (Figure 3). The parameter that exhibited the biggest area of the area under the curve (AUC) was selected to determine the cutoff values. According to the criteria, tamoxifen treatment and clinical stages were selected to determine the cutoff values for Ano1 and Ki67, respectively. Based on these results, a cutoff score of 65% and 38% were determined for the expression of Ano1 and Ki67, respectively. Immunohistological scores >65% and ≤65% were defined as the ‘high” and “low” expression of Ano1, respectively. Immunohistological scores >38% and ≤ 38% were defined as the “high” and “low” expression of Ki67, respectively.

Figure 1 shows the representative immunohis-tochemical staining for the high and low expression of Ano1 and Ki67 in breast cancer samples. Of the 407 breast cancer samples, 175 (43%) samples exhibited the low expression of Ano1, and 232 (57%) samples showed the high expression of Ano1. 353 (86.7%) samples exhibited the low expression of Ki67, and 54 (13.3%) samples showed the high expression of Ki67.

### Association of Ano1 expression with Ki67 expression

We performed Spearman’s rank correlation coefficient analysis to analyze the association between the expression of Ano1 and Ki67 in breast cancer patients. Ano1 expression was negatively correlated with Ki67 expression (r=-0.99, *P*=0.045) (Table 2). Ano1 expression was significantly higher in ER-positive (*P*=0.048) or HER2-negative (*P*=0.040) patients with the low expression of Ki67 compared with those with the high expression of Ki67 (Table 3).

**Table 2:** The correlation analysis of Ano1 expression and Ki67 expression in patients with breast cancer

Parameter	Ano1 expression		*P*^†,‡^	OR (95%CI)^§^	Correlation coefficient^Л^
	Low n (%)	High n (%)			
**Ki67 expression**					
Low	145(41.1)	208(58.9)	**0.045**^†^	1 (reference)	-0.99
High	30(55.6)	24(44.4)	**0.047**^‡^	**0.558 (0.313-0.993)**	

^†^ *P* values were calculated from 2-sided chi-square tests or Fisher’s Exact Test.

^‡^*P* values were calculated by unconditional logistic regression adjusted for age, menopause state.

§OR and 95% CI values were calculated by unconditional logistic regression adjusted for age, menopause status, first degree family history of breast cancer.

^Л^correlation coefficient was calculated by using Spearman’s rank correlation coefficient analysis.

**Table 3:** Association of Ano1 expression with Ki67 expression in breast cancer patients with different ER, PR, and HER2 status

Ki67 expression	Ano1 expression			
	Low n (%)	High n (%)	*P* value^†,‡^	OR(95%CI)^§^
**ER-positive patients**				
Low	97(39.9)	146(60.1)	0.054^†^	1 (reference)
High	20(57.1)	15(42.9)	**0.048**^‡^	**0.480(0.232-0.995)**
**ER-negative patients**				
Low	48(43.6)	62(56.4)	0.467^†^	1 (reference)
High	10(52.6)	9(47.4)	0.480^‡^	0.699(0.259-1.885)
**PR-positive patients**				
Low	94(39.2)	146(60.8)	0.289^†^	1 (reference)
High	17(48.6)	18(51.4)	0.250^‡^	0.654(0.317-1.348)
**PR-negative patients**				
Low	51(45.1)	62(54.9)	0.060^†^	1 (reference)
High	13(68.4)	6(31.6)	0.057^‡^	0.356(0.123-1.029)
**HER2-postive patients**				
Low	25(29.8)	59(70.2)	0.801^†^	1 (reference)
High	4(33.3)	8(66.7)	0.935^‡^	0.942(0.227-3.911)
**HER2-negative patients**				
Low	120(44.6)	149(55.4)	**0.037**^†^	1 (reference)
High	26(61.9)	16(38.1)	**0.040**^‡^	**0.497(0.255-0.969)**

**Abbreviations:** ER, Estrogen receptor; PR, Progesterone receptor; HER2, Human epidermal growth factor receptor.

^†^ *P* values were calculated from 2-sided chi-square tests or Fisher’s Exact Test.

^‡^*P* values were calculated by unconditional logistic regression adjusted for age, menopause state.

^§^OR and 95% CI values were calculated by unconditional logistic regression adjusted for age, menopause status, first degree family history of breast cancer.

### Association of the expression of Ano1 and Ki67 with the survival of breast cancer patients

**Figure 4:**
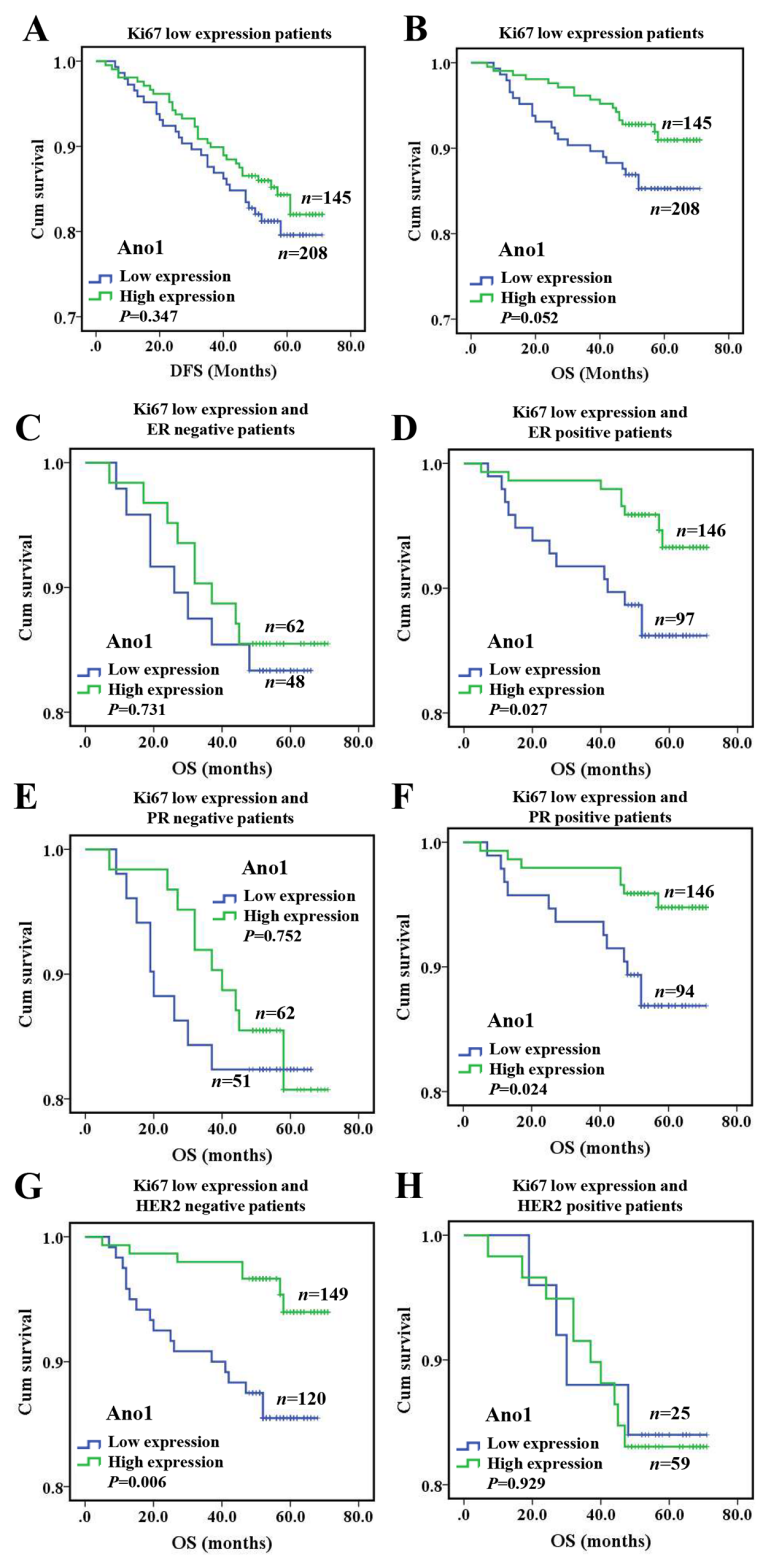
Kaplan-Meier survival analysis of Ano1 expression in breast cancer patients with the low expression of Ki67 **(A, B)** Survival curves show the association between Ano1 expression and DFS (A) and OS (B) in 353 breast cancer patients with the low expression of Ki67. C-H. Survival curves show the association between Ano1 expression and OS in ER-negative patients **(C)**, ER-positive patients **(D)**, PR-negative patients **(E)**, PR-positive patients **(F)**, HER2-negative patients **(G)**, and HER2-positive patients **(H)**.

We then evaluated the association of the expression of Ano1 and Ki67 with the OS or DFS in breast cancer patients. Ano1 expression was not associated with DFS or OS in breast cancer with the high expression of Ki67, regardless of the ER, PR, and HER2 status (Supplementary Figure 1). The high expression of Ano1 was associated with a tendency toward a longer OS in breast cancer with the low expression of Ki67 (*P*= 0.052, Figure 4B), especially in ER-positive (*P*=0.027, Figure 4D), PR-positive (*P*=0.024, Figure 4F), or HER2-negative (*P*=0.006) breast cancer (Figure 4G). Multivariate Cox regression analysis showed that Ano1 overexpression was a prognostic factor for longer OS in ER-positive, PR-positive or HER2-negative patients with the low expression of Ki67 (Table 4)

**Table 4:** Multivariate COX regression analysis of the association of Ano1 and Ki67 expression with DFS and OS in breast cancer patients

Variable	DFS				OS			
	Total n.	Events n (%)	Adjusted HR (95%CI)	*P* value^†^	Total *n*	Events n (%)	Adjusted HR (95%CI)	*P* value^†^
**Ano1 Expression**								
**ER-positive patients**								
Low	117	7 (6.0)	1 (reference)		117	16 (13.7)	1 (reference)	
High	161	19 (11.8)	1.869(0.780-4.480)	0.161	161	12 (7.5)	0.503(0.236-1.070)	0.074
**ER-negative patients**								
Low	58	6 (10.3)	1 (reference)		58	10 (17.2)	1 (reference)	
High	71	5 (7.0)	0.561(0.164-1.913)	0.356	71	11 (15.5)	0.938(0.391-2.252)	0.887
**PR-positive patients**								
Low	111	10 (9.0)	1 (reference)		111	16 (14.4)	1 (reference)	
High	164	16 (9.8)	1.015(0.455-2.262)	0.971	164	11 (6.7)	**0.425(0.196-0.921)**	**0.030**
**PR-negative patients**								
Low	64	3 (4.7)	1 (reference)		64	10 (15.6)	1 (reference)	
High	68	8 (11.8)	2.065(0.536-7.961)	0.292	68	12 (17.6)	1.087(0.465-2.540)	0.848
**HER2-positive patients**								
Low	29	3 (10.3)	1 (reference)		29	5(17.2)	1 (reference)	
High	67	8 (11.9)	0.955(0.240-3.805)	0.948	67	13(19.4)	1.498(0.489-4.589)	0.479
**HER2-negative patients**								
Low	285	149 (52.3)	1 (reference)		280	155(55.4)	1 (reference)	
High	26	16 (61.5)	1.256(0.564-2.794)	0.577	31	10(32.3)	**0.388(0.182-0.826)**	**0.014**
**Patients with the low expression of Ki67**								
Low	106	8(7.5)	1 (reference)		106	14(13.2)	1 (reference)	
High	169	19(11.2)	1.226(0.585-2.570)	0.590	169	9(5.3)	0.536(0.282-1.019)	0.057
**Patients with the high expression of Ki67**								
Low	22	2(9.1)	1 (reference)		22	4(18.2)	1 (reference)	
High	19	2(10.5)	2.146(0.334-13.785)	0.421	19	5(26.3)	1.569(0.445-5.526)	0.483
**Lymph node-negativepatients**								
Low	79	11(13.9)	1 (reference)		79	5(6.3)	1 (reference)	
High	120	14(11.7)	0.783(0.351-1.750)	0.551	120	5(4.2)	0.533(0.151-1.877)	0.327
**Lymph node-positivepatients**								
Low	96	23(24.0)	1 (reference)		96	21(21.9)	1 (reference)	
High	112	26(23.2)	0.975(0.546-1.678)	0.879	112	18(16.1)	0.694(0.370-1.303)	0.256
**ER-negativepatients with the low expression of Ki67**								
Low	48	11(22.9)	1 (reference)		48	8(16.7)	1 (reference)	
High	62	12(19.4)	0.870(0.376-2.015)	0.746	62	9(14.5)	0.900(0.340-2.385)	0.832
**ER-positive patients with the low expression of Ki67**								
Low	97	17(17.5)	1 (reference)		97	13(13.4)	1 (reference)	
High	146	21(14.4)	0.722(0.407-1.468)	0.431	146	8(5.5)	**0.374(0.155-0.906)**	**0.029**
**PR-negative patients with the low expression of Ki67**								
Low	51	11(21.6)	1 (reference)		51	9(17.6)	1 (reference)	
High	62	15(24.2)	1.082(0.492-2.379)	0.844	62	10(16.1)	0.880(0.353-2.194)	0.880
**PR-positive patients with the low expression of Ki67**								
Low	94	17(18.1)	1 (reference)		94	12(12.8)	1 (reference)	
High	146	18(12.3)	0.627(0.320-1.227)	0.173	146	7(4.8)	**0.328(0.128-0.841)**	**0.020**
**HER2-negative patients with the low expression of Ki67**								
Low	120	22(18.3)	1 (reference)		120	17(14.2)	1 (reference)	
High	149	17(12.1)	0.602(0.321-1.127)	0.113	149	7(4.7)	**0.296(0.122-0.717)**	**0.007**
**HER2-positive patients with the low expression of Ki67**								
Low	25	6(24.0)	1 (reference)		25	4(16.0)	1 (reference)	
High	59	15(25.4)	1.212(0.456-3.225)	0.700	59	10(16.9)	1.402(0.420-4.680)	0.583
**Ki67 Expression**								
**Lymph node-negative patients**								
Low	177	23(13.0)	1 (reference)		177	9(5.1)	1 (reference)	
High	22	2(9.1)	0.667(0.155-2.861)	0.585	22	1(4.1)	0.831(0.104-6.641)	0.861
**Lymph node-positive patients**								
Low	176	38(21.6)	1 (reference)		176	29(16.5)	1 (reference)	
High	32	11(34.4)	0.551(0.280-1.087)	0.085	32	10(31.3)	**0.482(0.233-0.995)**	**0.048**
**Lymph node-positive patients with the high expression of Ano1**								
Low	98	20(20.4)	1 (reference)		98	13(13.3)	1 (reference)	
High	14	6(42.9)	**2.649(1.060-6.617)**	**0.037**	14	5(35.7)	**3.286(1.170-9.228)**	**0.024**
**Lymph node-positive patients with the low expression of Ano1**								
Low	78	18(23.1)	1 (reference)		78	16(20.5)	1 (reference)	
High	18	5(27.8)	1.262(0.468-3.400)	0.646	18	5(27.8)	1.453(0.532-3.970)	0.466

**Abbreviations: HR**: Hazard Ratio; **95% CI**, 95% confidence interval; **DFS**: Disease-free survival; **OS**: Overall survival; Ref, reference category; **ER**, Estrogen receptor; **PR**, Progesterone receptor; **HER2**, Human epidermal growth factor receptor.

^†^*P* values, Adjusted HR(95%CI) were assessed using multivariate Cox regression analysis adjusted for age and menopausal status.

**Figure 5:**
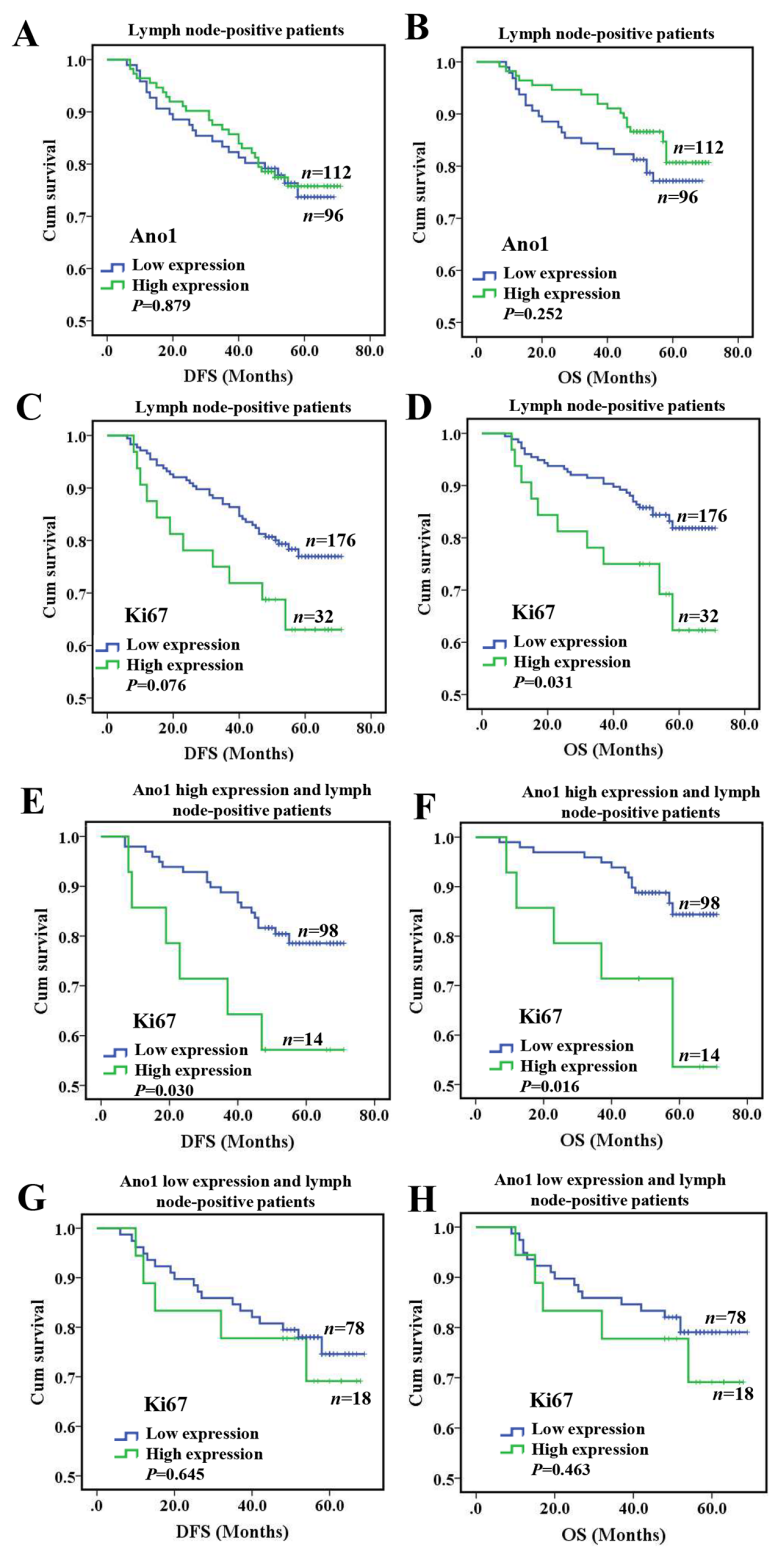
Kaplan-Meier survival analysis of Ano1 or Ki67 expression in breast cancer patients with lymph node metastasis **(A-D)** Survival curves show the association of the expression of Ano1 (A, B) and Ki67 (C, D) with DFS (A, C) and OS (B, D) in 208 lymph node-positive breast cancer patients. **(E-H)** Survival curves show the association between the expression of Ki67 with DFS (E, G) and OS (F, H) in lymph node-positive breast cancer patients with the high (E, F) and low (G, H) expression of Ano1.

We then investigated the association of the expression of Ano1 and Ki67 with the OS or DFS in breast cancer patients with lymph node-positive or node-negative status. In lymph node-negative patients, the expression of Ano1 or Ki67 was not significantly associated with the DFS or OS (Supplementary Figure 2A-2D). In lymph node-positive patients, Ano1 expression was not significantly associated with the DFS (*P*=0.879) or OS (*P*=0.252) (Figure 5A, 5B). In node-positive patients, Ki67 expression was significantly associated with shorter OS (*P*=0.031, Figure 5D), but not DFS (*P*=0.076, Figure 5C). Furthermore, in lymph node-positive patients with the high expression of Ano1, Ki67 expression was significantly associated with shorter DFS (*P*=0.030, Figure 5E) and OS (*P*=0.016, Figure 5F). However, no significant association was found between Ki67 expression with DFS and OS in lymph node-positive patients with the low expression of Ano1 (Figure 5G, 5H). Multivariate Cox regression analysis showed that Ki67 overexpression was a prognostic factor for shorter OS in lymph node-positive patients with the high expression of Ano1 (Table 4).

### Differential regulation of cell proliferation by Ano1 in MCF7 and MDA-MB-435S cells

**Figure 6:**
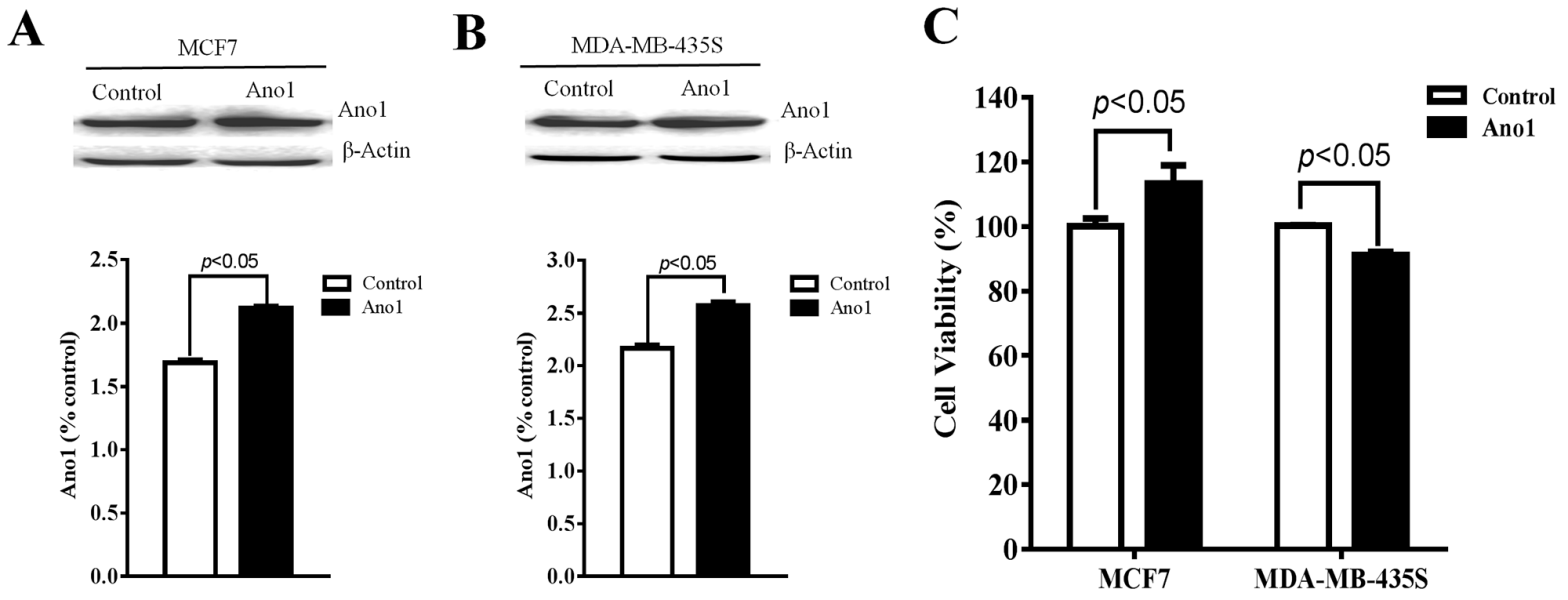
Regulation of cell proliferation by Ano1 **(A, and B)** Representative western blot showing the expression of Ano1 in MCF7 cells (A) or MDA-MB-435S cells (B) transfected with Ano1 plasmids or empty vectors. b-actin was used as a loading control. **(C)** CCK-8 assay showing the cell viability in MCF7 cells or MDA-MB-435S cells transfected with Ano1 plasmid or empty vectors. *p<0.05. n=3.

**Figure 7:**
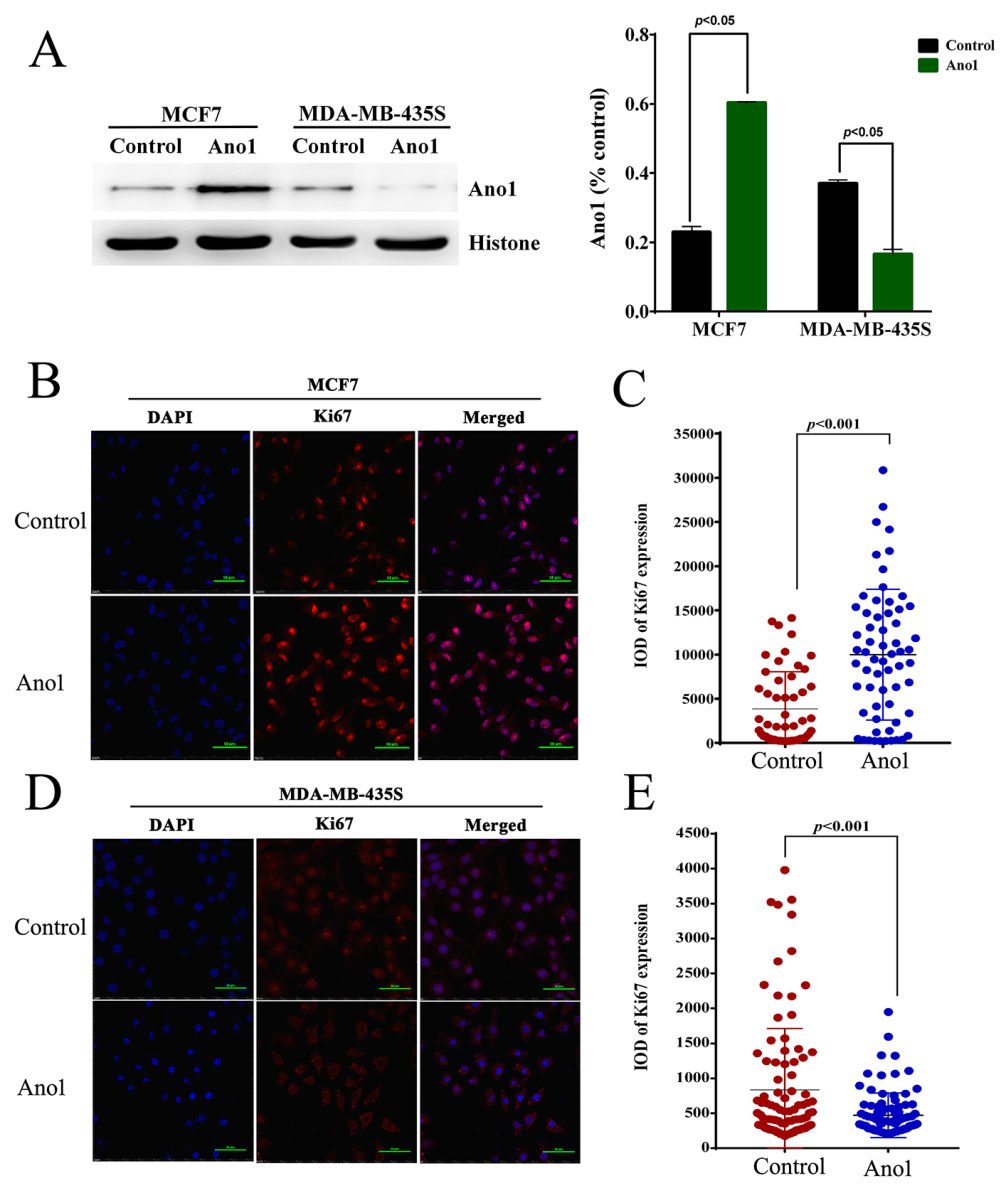
Ano1 overexpression regulates the nuclear expression of Ki67 **(A)** Representative Western blot showing the nuclear expression of Ki67 in MCF7 cells or MDA-MB-435S cells transfected with Ano1 plasmids or empty vectors (control). Histone was used as a loading control. **(B-E)** Representative immunofluorescent staining for Ano1 and Ki67 in MCF7 **(B)** or MDA-MB-435S **(D)** cells transfected with Ano1 plasmids or empty vectors. Scatter plots analysis of the IOD values of Ki67 expression in MCF7 **(C)** or MDA-MB-435S **(E)** cells transfected with Ano1 plasmids or empty vectors. *p<0.05. *n*=3.

**Figure 8:**
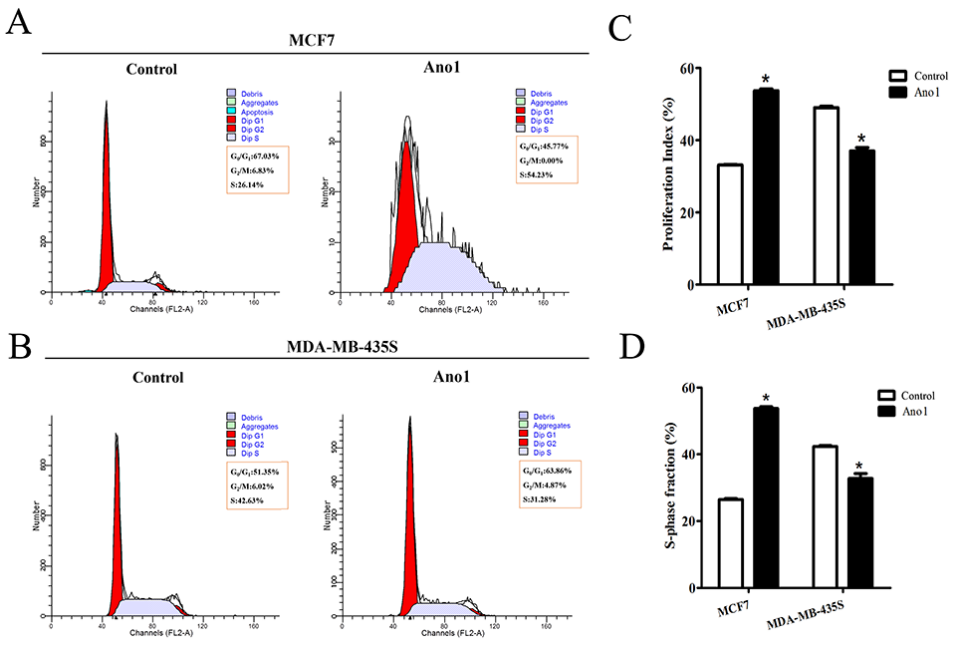
Effect of Ano1 on cell cycle **(A** and **B)**, Representative Flow cytometry for cell cycle analysis in MCF7 **(A)** or MDA-MB-435S **(B)** cells transfected with Ano1 plasmids or empty vectors. **(C** and **D)**, Proliferation index **(C)** and S-phase fraction **(D)** in MCF7 and MDA-MB-435S cells transfected with Ano1 plasmids or empty vectors. *p<0.05. *n*=3.

We then investigated whether Ano1 differentially regulated cell proliferation in different cells with different ER, PR, and HER2 status. Western blot analysis showed that Ano1 was expressed in MCF7 cells with ER-positive, PR-positive and HER2-negative status and MDA-MB-435S cells with ER-negative, PR-negative and HER2-negative status. Transfection of Ano1 plasmids resulted in a significant increase in Ano1 expression (Figure 6A, 6B). Ano1 overexpression resulted in a significant increase in cell viability in MCF7 cells and a significant decrease in cell viability in MDA-MB-435S cells (Figure 6C). In addition, Western blot analysis showed that Ano1 overexpression increased the nuclear expression of Ki67 in MCF7 cells, and inhibited the nuclear expression of Ki67 in MDA-MB-435S cells (Figure 7A). Consistent with Western blot results, immunofluorescence analysis showed that Ano1 overexpression significantly increased Ki67 expression in MCF7 cells, and significantly decreased Ki67 expression in MDA-MB-435S cells (Figure 7B–7E), suggesting that Ano1 regulated cell proliferation in a cell-specific manner. Furthermore, cell cycle analysis showed that Ano1 overexpression increased the percentage of MCF7 cells in the S phase, but decreased the percentage of MDA-MB-435S cells in the S phase (Figure 8). Taken together, these findings showed that Ano1 overexpression promoted cell proliferation in MCF7 cells, and inhibited cell proliferation in MDA-MB-435S cells.

## DISCUSSION

We and others have previously reported that Ano1 is overexpressed in breast cancer [18, 19]. However, it is unclear how Ano1 overexpression contributes to breast cancer tumorigenesis, and there are conflicting results regarding the role of Ano1 in cell proliferation in different cells [17, 18, 30, 34]. Breast cancer is a heterogeneous disease with respect to cellular origin, cellular subtypes, genetic alterations, and clinical outcomes [40]. We have previously found that Ano1 overexpression is associated with good prognosis in PR-positive or HER2-negative breast cancer patients following tamoxifen treatment [19]. The role of Ano1 in breast cancer tumorigenesis may be explained by a cell-type specific mechanism defined by different ER, PR and HER2 status. In the present study, we investigated the cell-specific mechanisms of Ano1 in breast cancer cell proliferation in breast cancer tumors and cell lines with different ER, PR and HER2 status. We found that in ER-positive or HER2-negative patients, Ano1 expression was significantly higher in tumors with the low expression of Ki67 than in tumors with the high expression of Ki67. Furthermore, *in vitro* studies showed that Ano1 increased cell proliferation in ER-positive, PR-positive, and HER2-negative MCF7 cells, but inhibited cell proliferation in ER-negative, PR-negative, and HER2-negative MDA-MB-435S cells. Our findings support the idea that Ano1 differentially regulates breast cancer cell proliferation in a cell-specific manner, depending on the ER, PR, and HER2 status.

In this study, we found that Ano1 overexpression promoted cell proliferation in MCF7 cells *in vitro*. Consistent with the proliferation-promoting effect of Ano1 in MCF7 cells, several *in vitro* studies have shown the proliferation-promoting effect of Ano1 in HNSCC, breast cancer, and prostate cancer [18, 20, 30]. However, in human breast cancer samples, we found that Ano1 expression was negatively correlated with Ki67 expression. In ER-positive or HER2-negative patients, Ano1 expression was significantly higher in tumors with the low expression of Ki67 than in tumors with the high expression of Ki67. It appears that Ano1 overexpression may inhibit cell proliferation in human breast cancer with ER-positive or HER2-negative status. These seemingly controversial results may be because the cellular condition may be different between *in vivo* human breast cancer tissues and *in vitro* cultured cells. Human breast cancer tissues contain heterogeneous cell populations, and it may be possible that a signaling pathway that promotes cell proliferation and inhibits Ano1 expression may be activated in breast cancer tissues, but not in cultured cells in our experimental conditions. Furthermore, we found that Ano1 overexpression inhibited cell proliferation in MDA-MB-435S cells with ER-negative, PR-negative, and HER2-negative status. It appears that the effect of Ano1 on cell proliferation is dependent on cellular conditions defined by ER, PR and HER2 status. The inhibitory effect of Ano1 on cell proliferation is supported by previous reports showing that Ano1 inhibited cell proliferation in vascular smooth muscle cells [34, 35]. The cell-specific effect of Ano1 on regulation of cell proliferation is further demonstrated by several studies showing that knockdown of Ano1 does not affect proliferation in HEK-293 cells [17], HNSCC BHY cells [25], colonic epithelial HT_29_ cells [26], pancreatic ductal adenocarcinoma cells [21], and gastric cancer cells [22].

The cell-specific mechanism underlying regulation of cell proliferation by Ano1 remains unclear, and may be associated with activation of different signaling pathways by Ano1 in different cancer cells. For example, in HNSSC, Ano1 activates ERK1/2 and increases the levels of cyclin D1 [30]. In gastric cancer, Ano1 promotes TGF-β secretion, and TGF-β signaling activation meditates Ano1-induced migration, invasion, and metastasis [22]. In glioma, Ano1 overexpression activates the nuclear factor-κB signaling pathway [31]. In breast cancer cell lines, Ano1 activates the epidermal growth factor receptor (EGFR) and calmodulin-dependent protein kinase (CAMK) signaling pathways [18]. In the present study, we found that Ano1 promoted cell proliferation in ER-positive, PR-positive, and HER2-negative MCF7 cells, but inhibited cell proliferation in ER-negative, PR-negative, and HER2-negative MDA-MB-435S cells. It appears that ER and PR signaling may determine the effect of Ano1 in cell proliferation. Further studies are required to identify the signaling pathways that are involved in the regulation of cell proliferation by Ano1 in MCF7 and MDA-MB-435S cells.

Cell lines exhibit a relatively high degree of homogeneity, whereas human breast cancer tissues are more heterogeneous. Therefore, the findings in one population of breast cancer cells may not represent the general feature of heterogeneous breast cancer tissues *in vivo*. In this study, we found a negative correlation between Ano1 and Ki67 in breast cancer samples, and the negative correlation was supported by Ano1 overexpression inhibited cell proliferation in MDA-MB-435S cells. However, although we found that Ano1 overexpression promoted cell proliferation in MCF7 cells, no positive correlation between Ano1 overexpression and Ki67 was found in human breast cancer samples. The proliferation-promoting effect of Ano1 in MCF7 cells may represent a specific feature of a certain breast cancer cell population and/or under a certain cellular condition. We found in lymph node-positive patients with the high, but not low, expression of Ano1, Ki67 overexpression was associated with a shorter OS. The poor prognosis of breast cancer patients with the high expression of Ano1 may be explained by the proliferation-promoting effect of Ano1 as identified in MCF7 cells.

Although it remains unclear whether Ano1 promotes or inhibits cell proliferation, there is a consistent finding of Ano1 overexpression in malignant cancers. Thus, Ano1 may be used as prognostic marker for cancer patients. It has been reported that Ano1 is associated with poor prognosis in patients with gastric cancer and HNSCC [22, 25]. Bristschgi et al. has found that Ano1 gene amplification is associated with poor prognosis in patients with breast cancer [18]. We have previously reported that Ano1 overexpression is associated with good prognosis in PR-positive or HER2-negative breast cancer patients following tamoxifen treatment [19]. In this study, multivariate Cox regression analysis showed that Ano1 overexpression was a prognostic for longer OS in ER-positive, PR-positive, or HER2-negative patients with the low expression of Ki67. According to the 13^th^ Gallen International Breast Cancer Conference (2013) Expert panel [41], endocrine therapy with tamoxifen is the most important intervention for the treatment of Luminal A breast cancer with ER-positive, PR-positive, HER2-negative status and the low expression of Ki67. Our study suggests that Ano1 expression in combination of clinical relevant markers ER, PR, HER2, and Ki67 is useful for predicting clinical outcomes of breast cancer patients, especially those with luminal A breast cancer, following tamoxifen treatment. Therefore, in the future, Ano1 expression could be used to help better identify Luminal A breast cancer patients with more sensitivity to tamoxifen treatment. Furthermore, we found that Ki67 overexpression was significantly associated with a shorter OS in lymph node-positive patients with the high expression of Ano1. Multivariate Cox regression analysis showed that Ki67 overexpression was a prognostic factor for shorter OS in lymph node-positive patients with the high expression of Ano1. These findings suggest that the expression of Ano1 and Ki67 may be used for predicting prognosis in breast cancer patients with lymph node metastasis.

In summary, Ano1 may inhibit or promote cell proliferation in different cells, suggesting that additional factors that regulate Ano1 expression and function may be involved in the cell-type specific mechanism. Since Ano1 expression is regulated by many ways such as genetic amplification [18], epigenetic regulation by HDAC [28] and promotor methylation [29], many signaling pathways can be potentially involved in regulation of Ano1 expression. Changes in Ano1 expression have been reported to determine whether cancer cells grow or migrate [29]. In the present study, we found that Ano1 was more associated with ER-positive, PR-positive, or HER2-negative patients with the low expression of Ki67, and Ano1 promoted cell proliferation in ER-positive, PR-positive, and HER2-negative MCF7 cells, but inhibited cell proliferation in ER-negative, PR-negative, and HER2-negative MDA-MB-435S cells. It appears that the ER, PR, and HER2 signaling pathways may affect the effect of Ano1 on cell proliferation. Ano1 has been found to activate different signaling pathways such as the ERK1/2 [30], TGF-β [22], nuclear factor-κB [31], EGFR and CAMK [18] signaling pathways. It remains to determine how certain pathways are activated in specific cells, and how activation of these signaling pathways affects the role of Ano1 in cell proliferation. Furthermore, we found that Ano1 overexpression was associated with longer OS in ER-positive, PR-positive or HER2-negative patients with the low expression of Ki67. These findings suggest Ano1 may be a potential marker for predicting clinical outcome in breast cancer subtypes defined by the ER, PR and HER2, and Ki67 status.

## MATERIALS AND METHODS

### Patients

The Medical Ethics Committee of China Medical University approved this retrospective study. Due to the retrospective nature of the study, the Ethics Committee waived the need of informed consent by the patients.

Human breast tissue samples were obtained from 407 female patients with sporadic breast cancer, who underwent surgery at the Department of Surgical Oncology and the Department of General Surgery at the First Hospital of China Medical University between January 2008 and December 2009. The diagnosis of breast cancer was histopathologically confirmed. The inclusion criteria for breast cancer patients were: 1) invasive ductal carcinoma (IDC) (n=396) and invasive lobular carcinoma (ILC) (n=11); 2) availability of complete clinical data and follow-up status; 3) breast cancer samples were collected for analysis; 4) breast cancer patients initially diagnosed at our hospital without a previous history of radiation therapy, chemotherapy, and hormonal therapy; and 5) patients who underwent surgical removal at our hospital and received postoperative chemotherapy and/or endocrine therapy. The exclusion criteria were: 1) incomplete clinical data; 2) ER, PR, and HER2 status were not tested, not recorded, or unknown; 3) HER2-positive status with 2+ immunostaining was not confirmed by fluorescent in situ hybridization (FISH); 4) patients who underwent postoperative radiation therapy; 5) patients who underwent adjuvant trastuzumab treatment; and 6) severe cardiovascular, pulmonary, renal, hepatic, and gastrointestinal diseases.

Clinicopathological data were retrospectively obtained from medical records. The following parameters were analyzed: patient age, menopausal status, family history, tumor size, histological grade, tumor stage, lymph node metastasis, ER status, PR status, HER2 status, and chemotherapeutic regimes. The histological grade of the cancer was determined according to the World Health Organization grading system. The stage of the cancer was evaluated according to the TNM staging system. ER, PR, and HER2 status were classified as positive and negative based on the immunohistochemistry (IHC) results in the medical records. ER-positive and PR-positive status were defined by >1% nuclear staining [37]. HER2 status was reported as follows: 0, 1+ for negative, 2+ for borderline, and 3+ for positive. HER2-immunostaining of 0-1+ and 3+ was defined as HER2-negative status and HER2-postive status, respectively. For tumors with HER2-immunostaining of 2+, FISH was performed to verify the positive status. HER2 gene amplification ratio of ≤2.2 and >2.2 by FISH was defined as HER2-negative, and HER2-postive status, respectively.

### Tissue microarray (TMA) and immunohistochemistry

Paraffin donor blocks containing representative breast cancer samples were selected by reviewing the hematoxylin and eosin-stained slides. Tissue cores with a diameter of 1.5 mm were extracted from each donor block, and precisely arrayed into a new paraffin recipient block with a maximum of 200 cores, using the Organization Microarrayer (Pathology Devices, USA). Sections (4 μm thick) were obtained from formalin-fixed and paraffin-embedded TMA blocks, mounted on poly-L-lysine-coated glass slides, and used for immunohistochemistry.

Immunohistochemistry was performed as previously described [19]. Briefly, sections were deparaffinized with xylene, rehydrated in a graded alcohol series, and washed in distilled water. Sections were then incubated in primary antibodies against Ano1 (Abcam Biotechnology, USA) or Ki67 (Abcam Biotechnology, USA) overnight at 4°C, followed by incubation with biotinylated secondary antibodies for 30 min at 37°C. The slides were then incubated with horseradish peroxidase-coupled streptavidin for additional 30 min (LSAB kit; Dako, Glostrup, Denmark), and stained with DAB (3, 3-diaminobenzidine). Sections were counterstained with hematoxylin, dehydrated, and mounted. Sections in which primary antibodies were replaced with normal rabbit IgG were used as negative controls.

### Evaluation of immunohistochemistry

The immunostaining was evaluated by two pathologists blinded to the experimental conditions. The intensity of immunoreactivity was scored as follows: 0 for no staining, 1 for weak staining, 2 for moderate staining, and 3 for strong staining. The proportion of tumor cells was calculated as the percentage of Ano1- or Ki67-immunopositive cells over the total tumor cells. Five sections were selected from each sample. For each section, five fields were randomly selected. Scores was assigned by using 5% increments (0%, 5%, 10%, …100%) as previously reported [19, 38, 39]. The average score for each sample was used for assessing cutoff score for overexpression of Ano1 or Ki67, using receiver operating characteristic curve (ROC). To generate ROC curves, the sensitivity and specificity for each outcome under study was plotted.

### Cell culture

The MCF7 and MDA-MB-435S cell lines were obtained from the American Type Culture Collection (ATCC, USA). The cells were maintained in Dulbecco’s Modified Eagle Medium (DMEM, Invitrogen, USA) containing 10% fetal bovine serum (HyClone, USA), 100 U/ml penicillin, and 100 mg/ml streptomycin in 5% CO_2_ in a humidified incubator at 37 °C. Cells were subcultured every 3 days, and used for the following experiments.

### Cell transfection

Plasmids containing Ano1 tagged with EGFP (enhanced green fluorescent protein) were kindly provided by Dr. U. Oh (Seoul National University, Korea). Ano1-overexpressing MCF7 or MDA-MB-435S cells were obtained by transfecting MCF7 or MDA-MB-435S cells with 1 μg Ano1 plasmids. Empty vectors were used as controls. After transfection for 48 h, cells were used for the following experiments.

### Western blot analysis

Cells were homogenized in ice-cold lysis buffer after cell transfection for 48 h. Proteins were resolved by SDS–PAGE, and transferred onto polyvinylidene fluoride membranes by electroblotting. Membranes were blocked with 5% milk, and then incubated with primary antibodies against Ano1 (1:3,000, Abcam Biotechnology, USA) or Ki67 (1:500, Abcam Biotechnology, USA) overnight at 4 °C. β-actin or histone was used as a loading control. Membranes were then incubated with horseradish peroxidase-conjugated goat anti-rabbit secondary antibodies (dilution 1:5,000, Abcam, USA) at room temperature for 1 h. Bands were visualized using an enhanced chemiluminescence detection system (Amersham, Freiburg, Germany).

### Cell Counting Kit-8 (CCK-8) assay

Cell viability was measured by using CCK-8 assay (Dojindo, JAPAN). After transfected with Ano1 or empty vectors, MCF7 or MDA-MB-435S cells (8,000 cells/well) were seeded into 96-well plates (Coring, Lowell, MA). Cells were allowed to grow in the growth medium for 48 h. Cells were then incubated with WST-8 (2-(2-methoxy-4-nitrophenyl)-3-(4-nitrophenyl)-5-(2,4-disulfophenyl)-2H-tetrazolium) for 2.5 h. Plates were read at 450 nm wavelength in a microplate reader (Anthos Labtec Instruments GmbH, Austria).

### Immunofluorescence

After transfected with Ano1 or empty vectors, cells were grown on glass coverslips in six-well plates until confluence. Cells were then rinsed with PBS, and fixed in 4% paraformaldehyde for 10 min at room temperature. Cells were permeabilized with 0.5% Triton-X-100 for 10 min, rinsed in PBS, and blocked with normal goat serum for 1 h at room temperature. Cells were then incubated with primary antibodies against Ki67 (Abcam Biotechnology, USA) (1:100 dilution) overnight at 4°C, followed by incubation with FITC-conjugated secondary antibodies (1:300 dilution; Invitrogen) for 1 h at room temperature. Cells were then counterstained with DAPI (4’,6- diamidino-2-phenylindole) for 15 min. The cells were examined and photographed under a confocal microscope (FV1000S-SIM/IX81, Olympus). Ki67-positive cells were defined as cells with the immunopositive staining in the nuclei. Integral optical density (IOP) was measured by using the IPP software (National Institutes of Health, USA).

### Flow cytometry

After transfected with Ano1 or empty vectors for 48 h, MCF7 or MDA-MB-435S cells were harvested and washed twice with cold PBS. Cells were then fixed with 70% ethanol and stored at 4 °C overnight. After rehydration with PBS, cells were treated with 20 μl (2 μg/ml) RNase A and incubated at 37 °C for 30 min. Cells were then stained with propidium iodide (PI, 50 μg/ml) for 1 h at 4 °C. Cells were analyzed for cell cycle by flow cytometry, using a MACSQuant instrument (Miltenyi, Germany).

### Statistical analysis

Analyses were performed using SPSS 16.0 (Chicago, IL, USA). Categorical data were compared using Pearson chi square tests or Fisher’s exact probability tests. Survival probabilities were estimated by the Kaplan-Meier method and assessed by a log-rank test. Multivariate Cox proportional hazards regression models was used for assessing the association between potential confounding variables and prognosis (overall survival (OS), or disease-free survival (DFS)). Probability values <0.05 were considered statistically significant.

## SUPPLEMENTARY MATERIALS FIGURES



## Abbreviations

Ano1: Anoctamin 1
OR: Odds ratio
CI: Confidence interval
DIC: Ductal invasive carcinoma
LIC: Lobular invasive carcinoma
ER: Estrogen receptor
PR: Progesterone receptor
HER2: Human epidermal growth factor receptor
Ki67: nuclear antigen Ki-67
