# Supplementary material for: Cell-specific regulation of proliferation by Ano1/TMEM16A in breast cancer with different ER, PR, and HER2 status
Source: Oncotarget. 2017 Jun 27;8(49):84996–5013. doi: 10.18632/oncotarget.18662 (PMC5689589; doi:10.18632/oncotarget.18662)
Supplement: Supplementary file 1 [file oncotarget-08-84996-s001.pdf]

Cell-specific regulation of proliferation by Ano1/TMEM16A in breast cancer with different ER, PR, and HER2 status

SUPPLEMENTARY MATERIALS

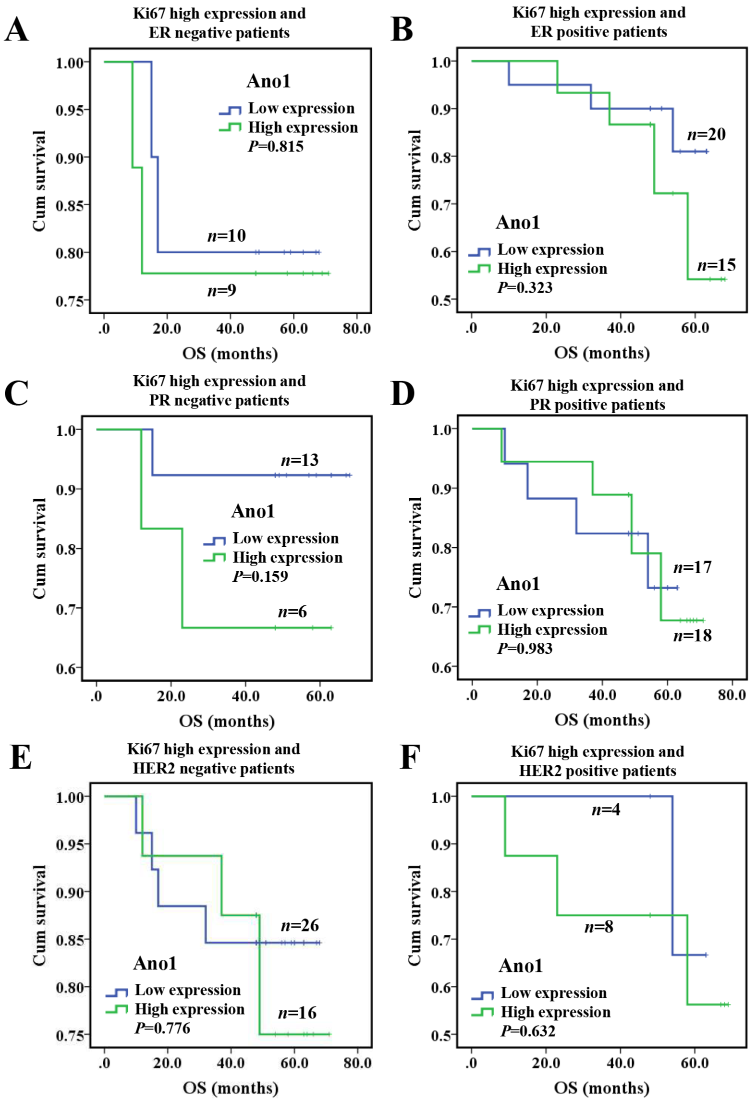

Supplementary Figure 1: Kaplan-Meier survival analysis of Ano1 expression in Ki67 high expression breast cancer patients with different ER, PR, and HER2 status. The log-rank test was performed to test the statistical significance. Survival curves show the association between Ano1 expression and OS in Ki67 high expression and ER-negative or -positive patients **(A, B)**, PR-negative or -positive patients **(C, D)**, and HER2-negative or -positive patients **(E, F)**.

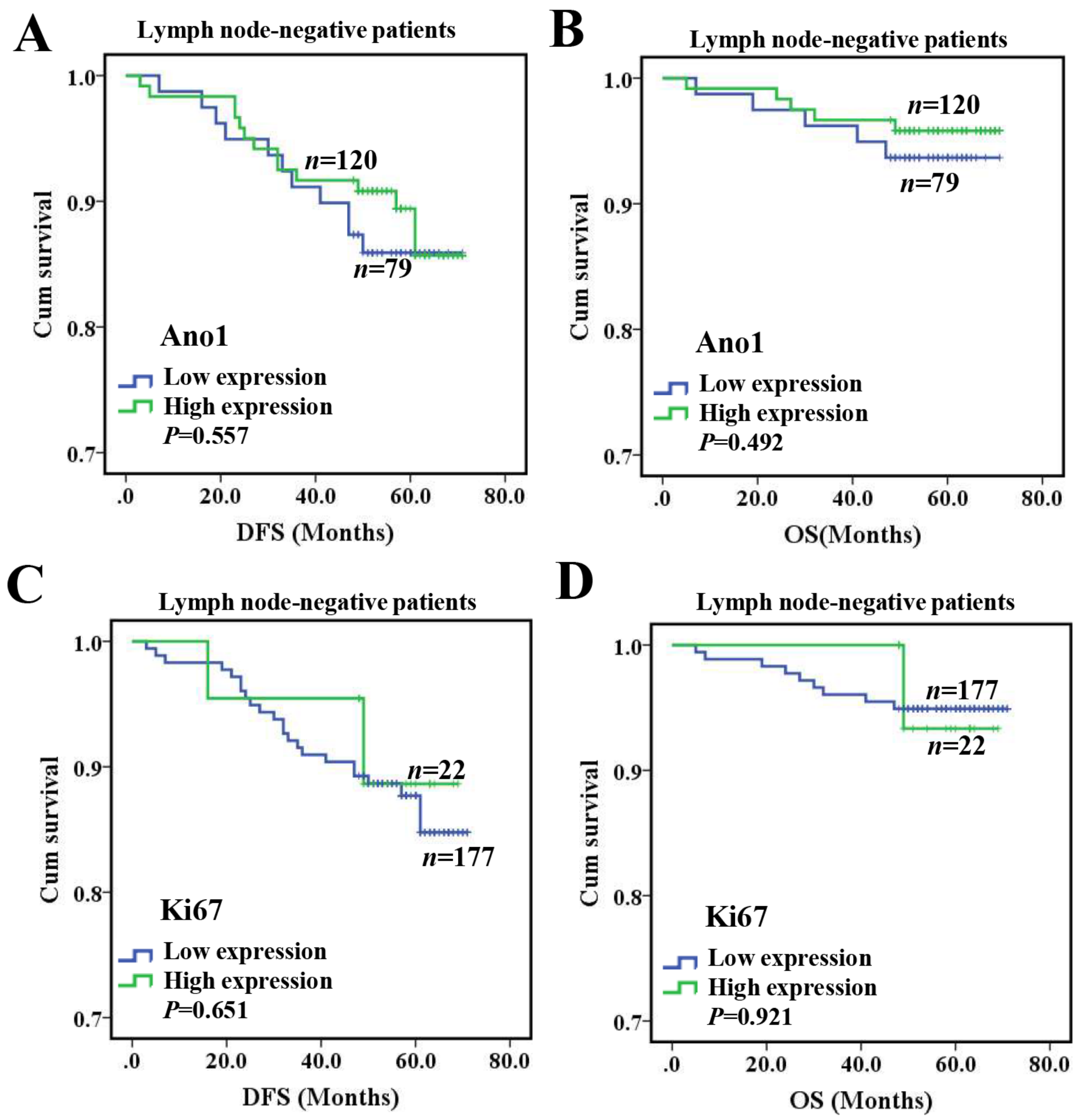

Supplementary Figure 2: Kaplan-Meier survival analysis of Ano1 or Ki67 expression in lymph node negative breast cancer patients. Survival curves show the association of the expression of Ano1 **(A, B)** and Ki67 **(C, D)** with DFS **(A, C)** and OS **(B, D)** in 199 lymph node-negative breast cancer patients.
